# Water quality assessment of Lugu Lake based on Nemerow pollution index method

**DOI:** 10.1038/s41598-022-17874-w

**Published:** 2022-08-10

**Authors:** Kai Su, Qin Wang, Linxiao Li, Rong Cao, Yingwei Xi

**Affiliations:** 1 Faculty of Geosciences and Environmental Engineering, Southwest Jiaotong University, Chengdu, 611756 China; 2 Department of Pollution Source and Emergency Monitoring, Sichuan Ecological Environment Monitoring Station, Chengdu, 610073 China

**Keywords:** Environmental impact, Environmental impact

## Abstract

In this paper, three monitoring sections were set up in Lugu Lake, and water samples were collected in 2019, 2020, and 2021 for the determination of physical and chemical properties such as permanganate index, chemical oxygen demand, biochemical oxygen demand (BOD_5_) and so on. By using the single factor pollution index method and the Nemerow pollution index method, the water quality of three monitoring sections and the whole Lugu Lake was assessed, and the temporal and spatial changes of water quality were analyzed. The findings demonstrate that Lugu Lake's overall water quality is excellent, and that it has not altered significantly in three years.The results of evaluating the water quality by the single factor pollution index method show that, in the past three years, the water quality of the three monitoring sections and the whole of Lugu Lake is Category I, which belongs to no pollution, and the measured indicators all meet the water quality standard of Category I. It can be seen from the evaluation results of the Nemerow index method that the water quality pollution index of Lugu Lake is between 0.22 and 0.34 in the past three years and the water quality evaluation of Changdao Bay, Lake center, Zhaojia Bay and the whole are Category I standards in 2019, 2020 and 2021. In terms of time changes, the water quality of Lugu Lake has remained stable between 2019 and 2021, and the water quality has been good. From the perspective of spatial changes, in 2019 and 2020, the water quality in Lake center is better than the monitoring sections of Changdao Bay and Zhaojia Bay.

## Introduction

As an important freshwater resource for human beings, lakes and reservoirs provide support for human life and production, maintain the ecological balance of the basin, and protect the local ecological environment^1^. A scientific method for evaluating water quality can yield an impartial assessment of the water environment's quality, assure the sustainable use of water resources, and serve as a foundation for environmental management and decision-making^2^. Therefore, scientific evaluation of lake and reservoir water quality is beneficial to the development, utilization and protection of lake and reservoir resources.

At present, the common methods of water environment quality evaluation mainly include single factor index method^3^, Nemerow pollution index method^4^, comprehensive pollution index method^5^, grey system evaluation method^6^, fuzzy comprehensive evaluation method^7^. The application of various water quality assessment methods in lakes and reservoirs has been relatively mature^8^. The pollution index approach, in comparison to other methods, is simple and conceptually clear, can quantitatively represent water quality, and the evaluation results can more accurately reflect the degree of pollution of water bodies^9^. For example, Liu and Chen^10^ used the Nemerow index method and principal component analysis method to comprehensively evaluate the groundwater environmental quality in the Honghu Lake area by combining qualitative analysis and quantitative analysis. Sun et al.^11^ applied the single factor pollution index method to obtain the main pollution factors and water quality categories of the rivers in the Hongdu Village section of the upper reaches of Jinxi, and combined the comprehensive pollution index to better reflect the pollution status of the entire river. Shan^12^ used the comprehensive water quality identification index method to analyze and evaluate the water quality at the entrance and exit of Shifosi Reservoir during the period from 2009 to 2015.

Lugu Lake is located at the junction of Yanyuan County in Sichuan Province and Ninglang County in Yunnan Province. The east of the lake is Lugu Lake Town, Yanyuan County, and the west of the lake is Yongning Township, Ninglang County. It is the third largest deep-water lake in China. As an important tourism resource, Lugu Lake has been rapidly developed and utilized. On the one hand, it has driven the local economic development, but at the same time, it has also brought certain damage to the lake and the surrounding ecological environment. The study of Lugu Lake primarily focuses on the ecological environment and changes in dissolved oxygen, but the study of other various monitoring indicators such as permanganate index and a variety of heavy metal ions is less monitored, so the single factor pollution index method and the Nemero pollution index method are used to study a number of indicators in order to provide new information. Therefore, this paper uses the water quality monitoring data of Lugu Lake from 2019 to 2021, adopts the single factor evaluation method and the Nemerow pollution index method to evaluate and analyze the water quality changes of Lugu Lake in three years, and discusses the impact of human activities on the water quality of Lugu Lake. In order to provide a scientific basis for the environmental protection of Lugu Lake and the sustainable development of water resources.

## Materials and method

### 1Research areas and data

**Figure 1:**
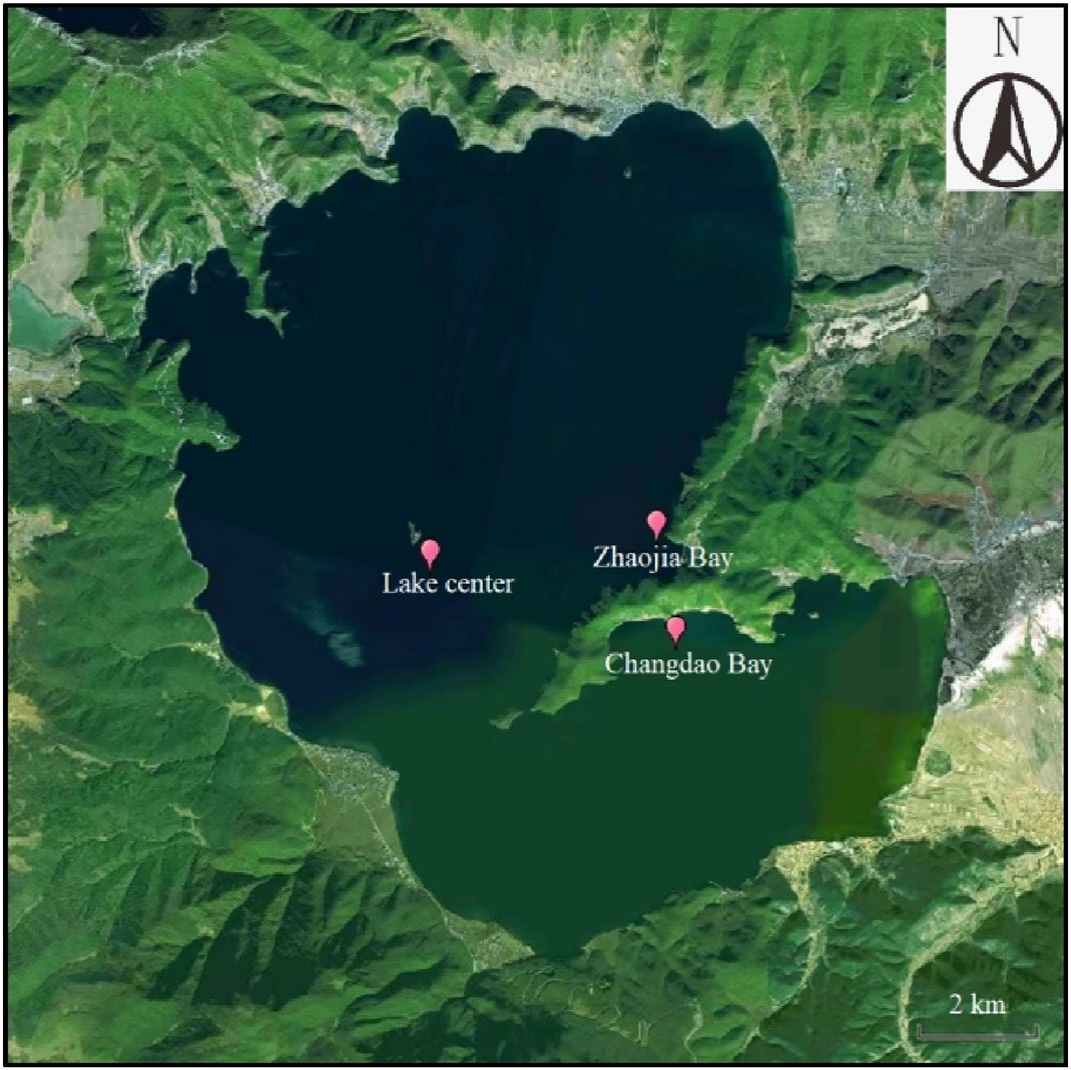
Sampling sites in Lugu Lake.

Lugu Lake (27°36'-27°47' N, 100°43'-100°54' E) is located in the hinterland of Hengduan Mountains at the junction of Ninglang County in Yunnan Province and Yanyuan County in Sichuan Province. It belongs to the Yalong River system. The elevation of the lake is 2690.8 m, the watershed area is 247.6 km^2^, the average water depth of the lake is 40.3 m, and the maximum water depth is 105.3 m^13^. The lake is 9.4 km long and 5.2 km wide^14^, with a catchment area of 187 km^2^ and a supply coefficient of 3.54. The lake's only outlet, Caohai, is located on the east coast. The water exchange cycle of the lake is 18.5 years, and it is a semi-closed lake^15^. The water quality of Lugu Lake was assessed using the annual average values of monitoring indicators in 2019 and 2020, as well as the average values of testing indicators from January to June 2021, for the Changdao Bay, Lake center, Zhaojia Bay, and the whole. The sample map of Lugu Lake is shown in Fig. 1.

### Research methods

#### Collection and determination of water samples

In this study, water samples were collected in 2019, 2020, and the first half of 2021 in accordance with the standards of the national surface water environmental quality monitoring network for collection, measurement and separation (on-site monitoring technical guidelines).Permanganate Index, COD, BOD_5_, TP, Ammonia Nitrogen, Mercury, Lead, Cadmium, Chromium (hexavalent), Arsenic, Copper, Selenium, Fluoride, Cyanide, Sulfide , petroleum (petroleum ether extraction) and volatile phenol of water samples was carried out in the laboratory according to the national standard method.

#### Water quality evaluation method

According to the research purpose, the single factor pollution index method and the Nemerow pollution index method were selected to comprehensively evaluate the water quality of the Lugu Lake in three years.

1. Single factor pollution index method

The comprehensive water quality category of the single factor index method is determined by comparing the water quality monitoring results with the relevant classification standards, that is, the category of the worst single index among all the indicators involved in the evaluation is used to determine the comprehensive water quality category of the water^16^. Through single factor evaluation, the main pollutants in the water and their hazard levels can be determined. The pollution index of the specific pollutant is calculated by dividing the actual measured value of the pollutant and the evaluation standard, and the evaluation result is expressed by the pollution index. The formula of the single factor pollution index method is:

1 $${\text{p}}_{{\text{i}}} = \frac{{{\text{C}}_{{\text{i}}} }}{{{\text{S}}_{{\text{i}}} }}$$

In the formula, Pi represents the pollution index of single water quality index i; Ci is the measured value of pollutant content (mg/L); S_i_ is the standard value of environmental quality (mg/L), which is the Category III water standard in the Environmental Quality Standards for Surface Water (GB3838-2002)^17^.

The environmental quality standard evaluation grading standard of the single factor pollution index method is shown in Table 1.

**Table 1:** Water quality level determination based on the single factor pollution index method.

Water quality level	P_i_	Pollution assessment
I	≤ 1	No pollution
II	(1,2]	Slightly pollution
III	(2,3]	Lightly pollution
IV	(3,5]	Moderately polluted
V	> 5	Seriously pollution

(2) Nemerow pollution index method

The Nemerow index is a weighted multi-factor environmental quality index that takes into account extreme values or outstanding maximum values^18^. The calculation method of the comprehensive pollution index is as follows:

2 $${\text{P}}_{{\text{N}}} = \sqrt {\frac{{\left( {{\text{P}}_{1} } \right)^{2} + {\text{P}}_{{{\text{imax}}}}^{2} }}{2}}$$

In the formula, P_N_ is the comprehensive pollution index of the sampling point; ${\mathrm{P}}_{\mathrm{imax}}$ is the maximum value of the single-item pollution index of the pollutants at the sampling point; ${\mathrm{P}}_{1}$=$\frac{1}{\mathrm{n}}\sum_{\mathrm{i}=1}^{\mathrm{n}}{\mathrm{P}}_{\mathrm{i}}$ is the average value of the single-factor index.

The grading standard for environmental quality evaluation by the Nemerow pollution index method^19^ is shown in Table 2.

**Table 2:** Water quality level determination based on the Nemerow pollution index method.

Water quality level	P_N_
I	< 0.59
II	[0.59,0.74)
III	[0.74,1.00)
IV	[1.00,3.50)
V	≥ 3.50

(3) Spatial and temporal variation characteristics of water quality in Lugu Lake.

The degree of water quality change with time and space is judged according to the time change rate T and the space change rate S of water quality, respectively. The calculation formula of T and S are as follows^20^,

3 $${\text{T}} = \frac{{G_{t1} - G_{t2} }}{{G_{t1} }} \times 100\%$$

4 $${\text{S}} = \frac{{G_{s1} - G_{s2} }}{{G_{s1} }} \times 100\%$$

In the formula, G_t1_ is the Nemerow index at the start time of the comparison time. G_t2_ is the Nemerow index of the sampling point at the termination time; G_S1_ and G_S2_ are the Nemerow index in the sampling points of the start section and the end section in the comparison space, respectively.

### Data processing

Excel was used for data processing, and Origin 2021 was used for graphing.

## Results and discussion

### Lugu Lake water quality index results

**Figure 2:**
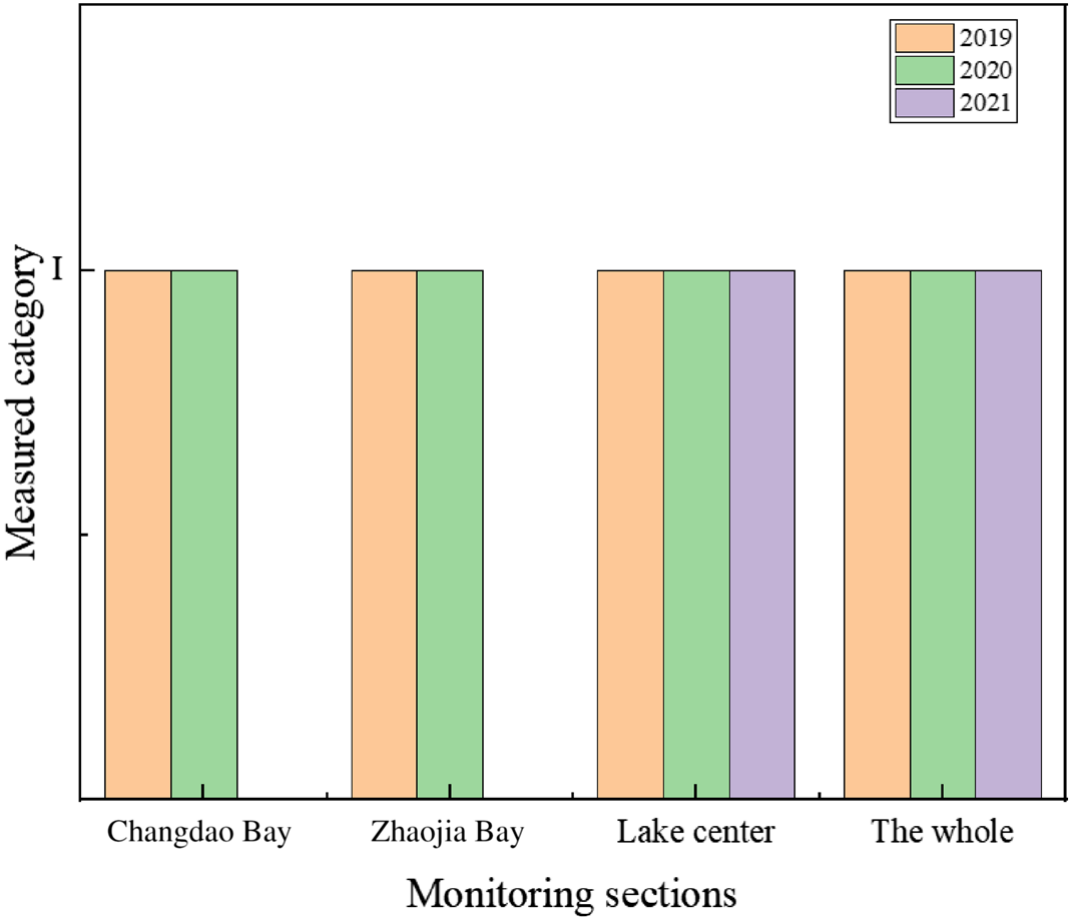
Water quality statistics of Lugu Lake in 2019, 2020 and 2021.

According to the Environmental Quality Standards for Surface Water (GB3838-2002), the water quality category of each monitoring section can be obtained by comparing the standard with each measured index. Tables 3, 4, and 5 exhibit the full results of the water quality index values of Lugu Lake in 2019, 2020, and 2021, respectively. And the statistical results of water quality categories of Lugu Lake in 2019, 2020 and 2021 are shown in Fig. 2.

**Table 3:** Statistical results of various indicators in Lugu Lake in 2019.

Monitoring section								
Monitoring indicators	Changdao Bay		Lake center		Zhaojia Bay		The whole	
	Average value	Mea-sured cate-gory	Average value	Mea-sured cate-gory	Average value	Mea-sured cate-gory	Average value	Mea-sured cate-gory
Permanganate Index	1.06 mg/L	I	1.20 mg/L	I	1.03 mg/L	I	1.09 mg/L	I
BOD_5_	600 µg/L	I	500 µg/L	I	500 µg/L	I	500 µg/L	I
Ammonia nitrogen	30 µg/L	I	20 µg/L	I	30 µg/L	I	30 µg/L	I
Petro	7 µg/L	I	5 µg/L	I	7 µg/L	I	6 µg/L	I
Volatile phenol	0.15 µg/L	I	0.20 µg/L	I	0.15 µg/L	I	0.17 µg/L	I
Hg	0.02 µg/L	I	0.02 µg/L	I	0.02 µg/L	I	0.02 µg/L	I
Pb	0.09 µg/L	I	0.90 µg/L	I	0.05 µg/L	I	0.34 µg/L	I
Cd	0.04 µg/L	I	0.08 µg/L	I	0.03 µg/L	I	0.05 µg/L	I
Anionic surfactant	25 µg/L	I	24 µg/L	I	25 µg/L	I	25 µg/L	I
Cr(hexavalent)	2 µg/L	I	2 µg/L	I	2 µg/L	I	2 µg/L	I
Fluoride(F^-^)	140 µg/L	I	156 µg/L	I	145 µg/L	I	147 µg/L	I
TP(P)	5 µg/L	I	5 µg/L	I	5 µg/L	I	5 µg/L	I
Cyanide	2 µg/L	I	1 µg/L	I	2 µg/L	I	2 µg/L	I
Sulfide	3 µg/L	I	3 µg/L	I	3 µg/L	I	3 µg/L	I
As	4.0 µg/L	I	3.2 µg/L	I	4.1 µg/L	I	3.7 µg/L	I
CODcr	8.92 mg/L	I	7.58 mg/L	I	7.42 mg/L	I	7.97 mg/L	I
Cu	0.4 µg/L	I	3.2 µg/L	I	1.7 µg/L	I	1.3 µg/L	I
Zn	10 µg/L	I	4 µg/L	I	5 µg/L	I	6 µg/L	I
Se(Tetravalent)	0.2 µg/L	I	0.4 µg/L	I	0.2 µg/L	I	0.3 µg/L	I

**Table 4:** Statistical results of various indicators in Lugu Lake in 2020.

Monitoring section								
Monitoring indicators	Changdao Bay		Lake center		Zhaojia Bay		The whole	
	Average value	Mea-sured cate-gory	Average value	Mea-sured cate-gory	Average value	Mea-sured cate-gory	Average value	Mea-sured cate-gory
Permanganate Index	1.26 mg/L	I	1.12 mg/L	I	1.30 mg/L	I	1.23 mg/L	I
BOD_5_	200 µg/L	I	500 µg/L	I	400 µg/L	I	400 µg/L	I
Ammonia nitrogen	40 µg/L	I	23 µg/L	I	48 µg/L	I	39 µg/L	I
Petro	9 µg/L	I	5 µg/L	I	8 µg/L	I	7 µg/L	I
Volatile phenol	0.15 µg/L	I	0.20 µg/L	I	0.15 µg/L	I	0.17 µg/L	I
Hg	0.02 µg/L	I	0.02 µg/L	I	0.02 µg/L	I	0.02 µg/L	I
Pb	0.05 µg/L	I	0.09 µg/L	I	0.05 µg/L	I	0.06 µg/L	I
Cd	0.03 µg/L	I	0.02 µg/L	I	0.03 µg/L	I	0.02 µg/L	I
Anionic surfactant	25 µg/L	I	20 µg/L	I	25 µg/L	I	23 µg/L	I
Cr(hexavalent)	2 µg/L	I	2 µg/L	I	2 µg/L	I	2 µg/L	I
Fluoride(F^-^)	180 µg/L	I	157 µg/L	I	180 µg/L	I	172 µg/L	I
TP(P)	6 µg/L	I	6 µg/L	I	6 µg/L	I	6 µg/L	I
Cyanide	2 µg/L	I	1 µg/L	I	2 µg/L	I	2 µg/L	I
Sulfide	3 µg/L	I	2 µg/L	I	3 µg/L	I	2 µg/L	I
As	4.6 µg/L	I	3.3 µg/L	I	4.6 µg/L	I	4.2 µg/L	I
CODcr	9.30 mg/L	I	6.13 mg/L	I	8.90 mg/L	I	8.11 mg/L	I
Cu	0.4 µg/L	I	0.4 µg/L	I	0.6 µg/L	I	0.5 µg/L	I
Zn	7 µg/L	I	1 µg/L	I	5 µg/L	I	4 µg/L	I
Se(Tetravalent)	0.2 µg/L	I	0.02 µg/L	I	0.2 µg/L	I	0.2 µg/L	I

**Table 5:** Statistical results of various indicators in Lugu Lake in 2021.

	Monitoring section			
Monitoring indicators	The whole		Lake center	
	Average value	Measured category	Average value	Measured category
Permanganate Index	1.37 mg/L	I	1.37 mg/L	I
BOD_5_	800 µg/L	I	800 µg/L	I
Ammonia nitrogen	30 µg/L	I	30 µg/L	I
Petro	5 µg/L	I	5 µg/L	I
Volatile phenol	0.2 µg/L	I	0.2 µg/L	I
Hg	0.04 µg/L	I	0.04 µg/L	I
Pb	0.17 µg/L	I	0.17 µg/L	I
Cd	0.02 µg/L	I	0.02 µg/L	I
Anionic surfactant	20 µg/L	I	20 µg/L	I
Cr(hexavalent)	2 µg/L	I	2 µg/L	I
Fluoride(F^-^)	184 µg/L	I	184 µg/L	I
TP(P)	7 µg/L	I	7 µg/L	I
Cyanide	0.5 µg/L	I	0.5 µg/L	I
Sulfide	2 µg/L	I	2 µg/L	I
As	4.6 µg/L	I	4.6 µg/L	I
CODcr	5.50 mg/L	I	5.50 mg/L	I
Cu	2 µg/L	I	2 µg/L	I
Zn	5.2 µg/L	I	5.2 µg/L	I
Se(Tetravalent)	0.2 µg/L	I	0.2 µg/L	I

From the data in the above three tables, it can be concluded that:

1. Comparing the average values of the water quality indicators of Lugu Lake with the standards in Environmental Quality Standards for Surface Water (GB3838-2002), it is found that: in 2019, 2020 and 2021, the water quality monitoring indicators of the three monitoring sections of Lugu Lake's Changdao Bay, Lake center and Zhaojia Bay all reached the Category I standard.
2. According to the data comparison for three consecutive years, it is found that the water quality of Lugu Lake is good, and the changes of various monitoring indicators are not large, which meets the requirements of surface water functional zoning. According to certain assessments, the Lugu Lake Nature Reserve's carrying capacity has reached a relatively saturated capacity in terms of space and tourism amenities, however the Lugu Lake Nature Reserve's water supply and sewage treatment capacity can still be enhanced^21^. As a result of the three years of data, the pollution generated by the Lugu Lake's surrounding environment can be discharged to the standard after treatment, allowing the lake's water quality to remain good and reasonably steady.

### Water quality evaluation and discussion

#### Evaluation of water quality by single factor pollution index method

According to the formula (1), the data of the four monitoring indicators in the Lugu Lake are calculated, and the single factor pollution index of each monitoring indicator in the Lugu Lake is shown in Table 6.

**Table 6:** Results of water quality assessment by single factor pollution index method.

Single factor pollution index, Pi										
Monitoring indicators	2019			2020			2021			
	Changdao Bay	Lake center	Zhaojia Bay	The whole	Changdao Bay	Lake center	Zhaojia Bay	The whole	Lake center	The whole
Permanganate Index	0.18	0.20	0.17	0.18	0.21	0.19	0.22	0.20	0.23	0.23
BOD_5_	0.15	0.13	0.13	0.13	0.05	0.13	0.10	0.10	0.20	0.20
Ammonia nitrogen	0.03	0.02	0.03	0.03	0.04	0.02	0.05	0.04	0.03	0.03
Petro	0.14	0.10	0.14	0.13	0.17	0.11	0.15	0.14	0.10	0.10
Volatile phenol	0.03	0.04	0.03	0.03	0.03	0.04	0.03	0.03	0.04	0.04
Hg	0.20	0.20	0.20	0.20	0.20	0.22	0.20	0.21	0.35	0.35
Pb	0.0018	0.0180	0.0009	0.0069	0.0009	0.0018	0.0009	0.0012	0.0034	0.0034
Cd	0.007	0.015	0.005	0.009	0.005	0.004	0.005	0.005	0.004	0.004
Anionic surfactant	0.13	0.12	0.13	0.12	0.13	0.10	0.13	0.12	0.10	0.10
Cr(hexavalent)	0.04	0.04	0.04	0.04	0.04	0.04	0.04	0.04	0.04	0.04
Fluoride(F^-^)	0.14	0.16	0.15	0.15	0.18	0.16	0.18	0.17	0.18	0.18
TP(P)	0.10	0.10	0.10	0.10	0.13	0.11	0.13	0.12	0.14	0.14
Cyanide	0.010	0.003	0.010	0.008	0.010	0.003	0.010	0.008	0.003	0.003
Sulfide	0.0125	0.0169	0.0125	0.0140	0.0125	0.0100	0.0125	0.0117	0.0100	0.0100
As	0.080	0.063	0.082	0.075	0.093	0.067	0.0912	0.0834	0.0920	0.0920
CODcr	0.45	0.38	0.37	0.40	0.47	0.31	0.45	0.41	0.28	0.28
Cu	0.0004	0.0032	0.0002	0.0013	0.0004	0.0004	0.0006	0.0005	0.0020	0.0020
Zn	0.010	0.004	0.005	0.006	0.007	0.001	0.005	0.004	0.005	0.005
Se(Tetravalent)	0.02	0.04	0.02	0.03	0.02	0.02	0.02	0.02	0.02	0.02

It can be seen from Table 6 that the single factor index method is based on the three types of water quality standards in the Environmental Quality Standards for Surface Water. In the 3 years of 2019, 2020 and 2021, the permanganate index, biochemical oxygen demand (BOD_5_), ammonia nitrogen, petroleum (petroleum ether extraction), volatile phenol, mercury, lead, cadmium, anionic surfactant, chromium, fluoride, total phosphorus, cyanide The water quality evaluation of sulfide, arsenic, CODcr, copper, zinc and selenium are all Category I standards.

Compared with the water quality standards in the Environmental Quality Standards for Surface Water, the single factor pollution evaluation method can more simply and intuitively reflect the pollution status of the water quality of Lugu Lake. The water quality of Lugu Lake is Category I standard, which is pollution-free.

#### Evaluation of water quality by Nemerow pollution index method

**Figure 3:**
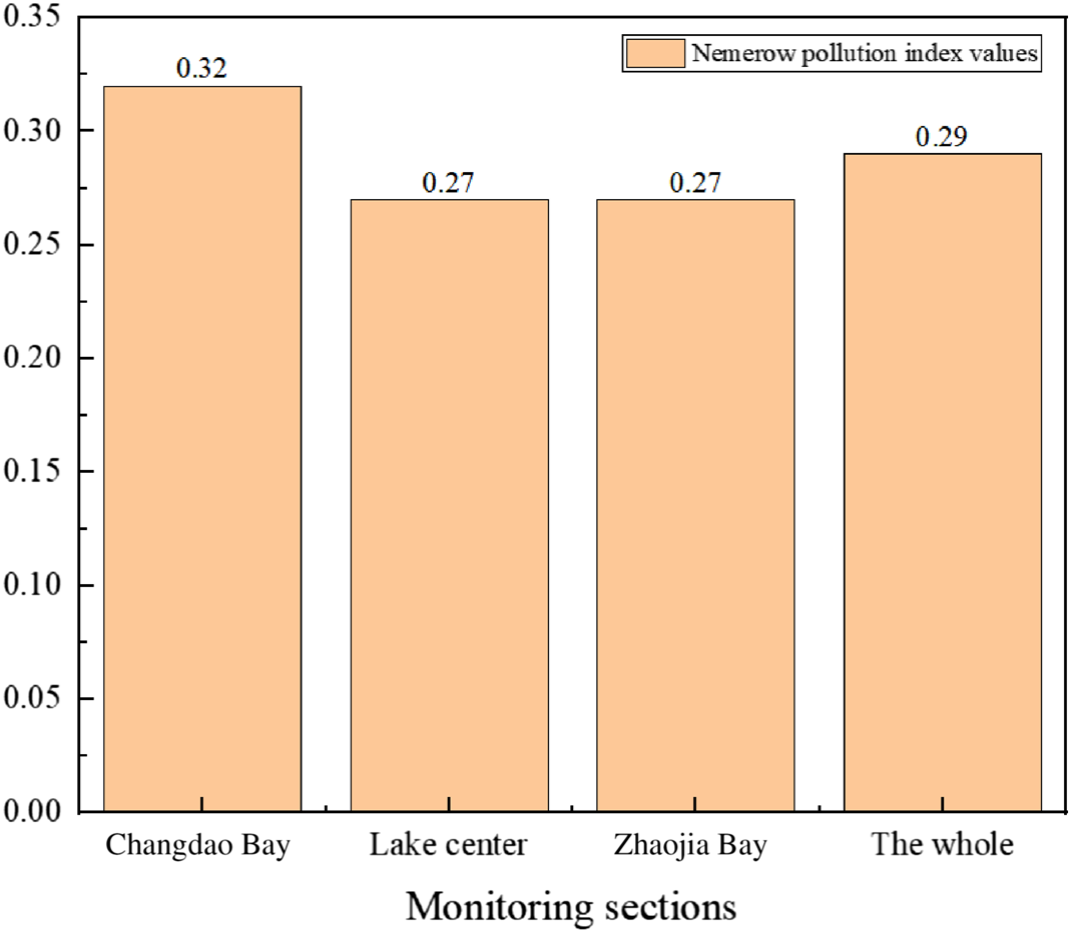
Comparison of Nemerow pollution index of various monitoring sections of Lugu Lake in 2019.

**Figure 4:**
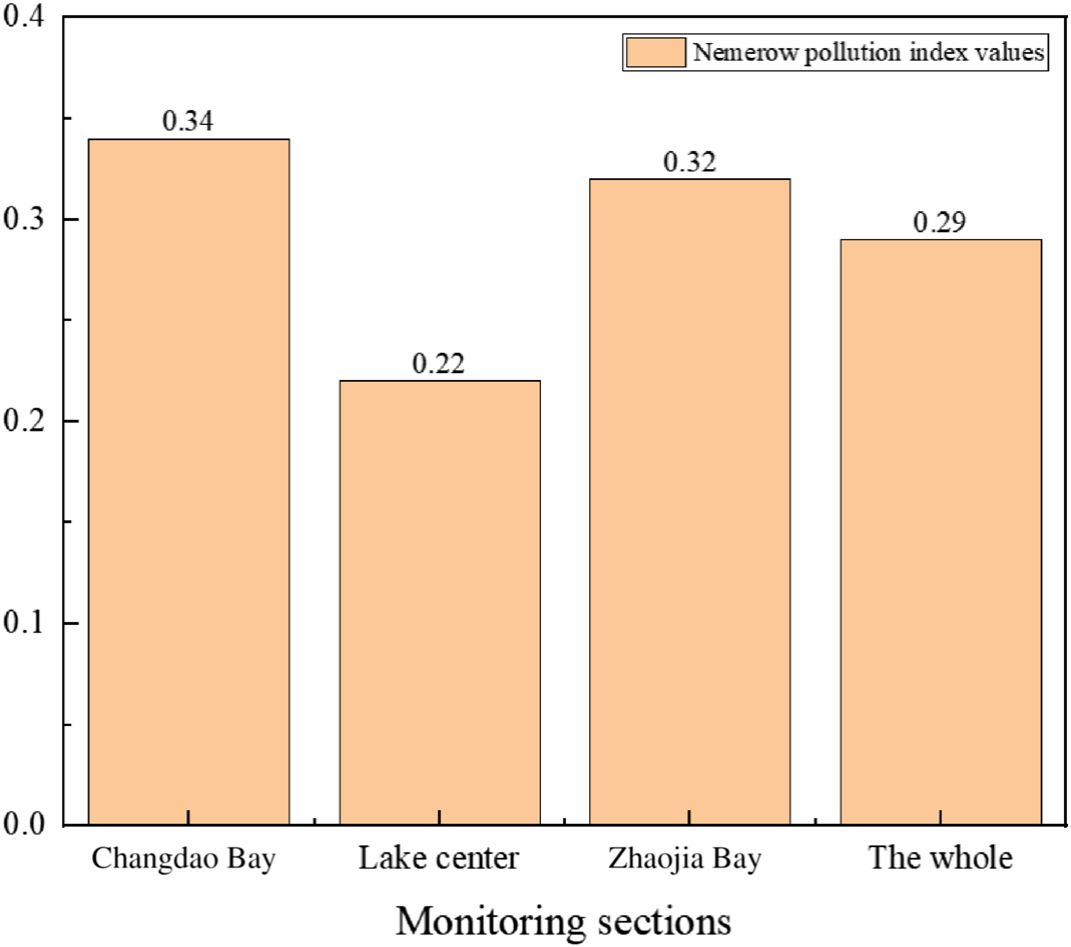
Comparison of Nemerow pollution index of various monitoring sections of Lugu Lake in 2020.

**Figure 5:**
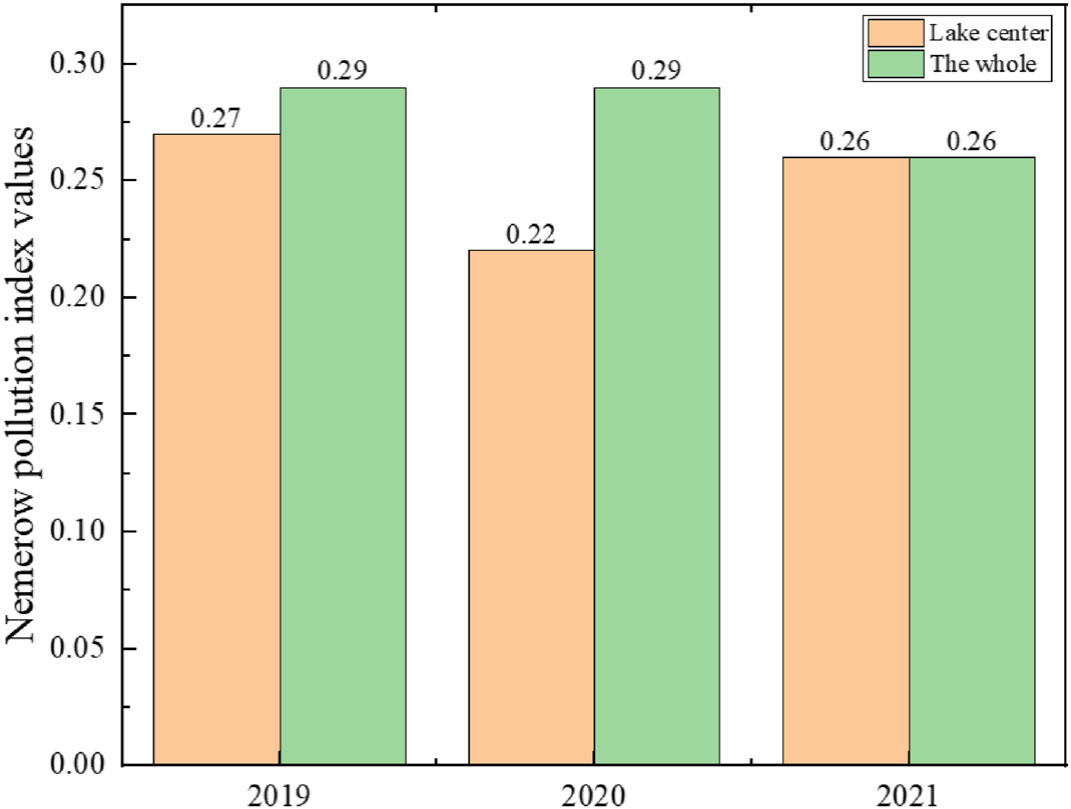
Changes in Nemerow pollution index for the Lake center and The whole from 2019 to 2021.

According to formula (2), the data of the four monitoring indicators in the Lugu Lake are calculated, and the Nemerow pollution index of each monitoring indicator in the Lugu Lake is shown in Figs. 3, 4 and 5.

The lower the Nemerow pollution index value, the higher the water body's quality. It can be seen from the evaluation results of the Nemerow index method that the water quality pollution index of Lugu Lake is between 0.22 and 0.34 in the past three years. The water quality of the three monitoring sections of Changdao Bay, Lake Center, and Zhaojia Bay did not change significantly, and the water quality of Lake Center has always been better than other monitoring sections. Throughout the three years, all of Lugu Lake's monitoring sections received a Category I water quality evaluation, indicating that the lake's water quality was exceptional. From the data in Fig. 5, it can be seen that the overall water quality of Lugu Lake has improved in the past three years, and it has always been classified as Category I water quality, which is clean.

Compared with the evaluation results of the water quality pollution status of each monitoring section of Lugu Lake by the single factor pollution index method, the Nemerow index method comprehensively considers the more seriously polluted indicators, which reflects the degree of water pollution, and also takes the average value of the single factor index^22^, making up for the shortcomings of the single factor index method, so the Nemerow index method is more comprehensive than the single factor index method for water quality evaluation.

#### Analysis of temporal and spatial variation characteristics of water quality

According to formulas (3) and (4), the temporal and spatial variation characteristics of water quality in each monitoring section of the Lugu Lake are calculated. The calculation results of the temporal and spatial variation rates are shown in Tables 7 and 8.

**Table 7:** Temporal and spatial changes of water quality in each monitoring section of Lugu Lake from 2019 to 2020.

Monitoring section	P_N_		T/%	S/%		Datum section
	2019	2020		2019	2020	
Changdao Bay	0.32	0.34	− 0.06	–	–	–
Lake center	0.27	0.22	0.19	0.16	0.35	Changdao Bay
Zhaojia Bay	0.27	0.32	− 0.19	0.00	− 0.31	Lake center
The whole	0.29	0.29	0.00	–	–	–

**Table 8:** Temporal and spatial changes of water quality in each monitoring section of Lugu Lake from 2020 to 2021.

Monitoring section	P_N_		T/%
	2020	2021	
Changdao Bay	0.34	–	–
Lake center	0.22	0.26	− 0.18
Zhaojia Bay	0.32	–	–
The whole	0.29	0.26	0.10

Three monitoring sections of Changdao Bay, Lake center and Zhaojia Bay were selected to analyze the temporal and spatial changes of water quality. According to Table 7, from 2019 to 2020, there was no significant change in the water quality of the lake center and the whole, and the water quality of Changdao Bay and Zhaojia Bay deteriorated slightly, but the change was not obvious. In 2019, the water quality of Changdao Bay was slightly worse than the rest of the monitoring sections, but the overall water quality was good. In 2020, the water quality of the monitoring sections of Changdao Bay and Zhaojia Bay was poor, and the water quality in Lake center was the best. According to Table 8, the water quality in the center of Lugu Lake in 2021 was lower than that in 2020, but the change was not obvious. The overall water quality is good.

## Conclusion

The overall water quality of Lugu Lake is good, and the water quality has not changed much in three years. In 2019, 2020 and 2021, it will meet the Category I water quality standard in the Environmental Quality Standards for Surface Water.

The results of evaluating the water quality by the single factor pollution index method show that, in the past three years, the water quality of the three monitoring sections and the whole of Lugu Lake is Category I, which belongs to no pollution, and the measured indicators all meet the water quality standard of Category I. It can be seen from the evaluation results of the Nemerow index method that the water quality pollution index of Lugu Lake is between 0.22 and 0.34 in the past three years and the water quality evaluation of Changdao Bay, Lake center, Zhaojia Bay and the whole are Category I standards in 2019, 2020 and 2021.

From the perspective of time changes, the water quality of Lugu Lake has not changed significantly from 2019 to 2021, and the water quality has been good. From the perspective of spatial changes, in 2019 and 2020, the water quality in Lake center is better than the monitoring sections of Changdao Bay and Zhaojia Bay.

## Data Availability

All data generated or analysed during this study are included in this published article.
